# Magnetic resonance diffusion kurtosis imaging in differential diagnosis of benign and malignant renal tumors

**DOI:** 10.1186/s40644-020-00369-0

**Published:** 2021-01-07

**Authors:** Jianxiong Fu, Jing Ye, Wenrong Zhu, Jingtao Wu, Wenxin Chen, Qingqiang Zhu

**Affiliations:** Department of Medical Imaging, Clinical Medical College, Yangzhou University, No 98 West Nantong Road, Yangzhou, 225001 China

**Keywords:** Differential diagnosis, Anisotropy, Renal neoplasms, Kidney, Diffusion magnetic resonance imaging

## Abstract

**Background:**

Benign and malignant renal tumors share similar some imaging findings.

**Methods:**

Sixty-six patients with clear cell renal cell carcinoma (CCRCC), 13 patients with renal angiomyolipoma with minimal fat (RAMF) and 7 patients with renal oncocytoma (RO) were examined. For diffusion kurtosis imaging (DKI), respiratory triggered echo-planar imaging sequences were acquired in axial plane (3 b-values: 0, 500, 1000s/mm^2^). Mean Diffusivity (MD), fractional Anisotropy (FA), mean kurtosis (MK), kurtosis anisotropy (KA) and radial kurtosis (RK) were performed.

**Results:**

For MD, a significant higher value was shown in CCRCC (3.08 ± 0.23) than the rest renal tumors (2.93 ± 0.30 for RO, 1.52 ± 0.24 for AML, *P* < 0.05). The MD values were higher for RO than for AML (2.93 ± 0.30 vs.1.52 ± 0.24, *P* < 0.05), while comparable MD values were found between CCRCC and RO (3.08 ± 0.23 vs. 2.93 ± 0.30, *P* > 0.05). For MK, KA and RK, a significant higher value was shown in AML (1.32 ± 0.16, 1.42 ± 0.23, 1.41 ± 0.29) than CCRCC (0.43 ± 0.08, 0.57 ± 0.16, 0.37 ± 0.11) and RO (0.81 ± 0.08, 0.86 ± 0.16, 0.69 ± 0.08) (*P* < 0.05). The MK, KA and RK values were higher for RO than for CCRCC (0.81 ± 0.08 vs. 0.43 ± 0.08, 0.86 ± 0.16 vs. 0.57 ± 0.16, 0.69 ± 0.08 vs. 0.37 ± 0.11, *P* < 0.05). Using MD values of 2.86 as the threshold value for differentiating CCRCC from RO and AML, the best result obtained had a sensitivity of 76.1%, specificity of 72.6%. Using MK, KA and RK values of 1.19,1.13 and 1.11 as the threshold value for differentiating AML from CCRCC and RO, the best result obtained had a sensitivity of 91.2, 86.7, 82.1%, and specificity of 86.7, 83.2, 72.8%.

**Conclusion:**

DKI can be used as another noninvasive biomarker for benign and malignant renal tumors’ differential diagnosis.

## Background

Diffusion tensor imaging (DTI) enables the diffusional motion of water molecules to be measured, providing a unique source of contrast among tissues [1, 2]. Because of structural hindrances in biological tissue like membranes or directional structures as in kidney, the diffusion of water molecules is restricted and does not follow a Gaussian distribution. To describe the diffusion process more correctly, mathematical models considering the deviation from the Gaussian behavior have been proposed [3].

Cheung et al. [4] compared the diffusivity values between diffusional kurtosis imaging (DKI) and a conventional DTI approach in rodent brain, with the outcome showing that DTI derived diffusivities were generally lower than those obtained by DKI. DKI provides different diffusion parameters, such as mean Diffusivity (MD), fractional Anisotropy (FA), mean kurtosis (MK), kurtosis anisotropy (KA) and radial kurtosis (RK). DKI can better reflect the microstructural complexity of tissue because it considers the non-Gaussian behavior of water in biological tissues [5, 6].

Recently [7, 8], DKI techniques have been used to investigate water diffusion in grading of cerebral gliomas, assessing aging-related changes in brain microstructure and showed a distinct signature for cerebrospinal fluid, grey matter and white matter. DKI was also successfully applied for the detection of ischemic stroke and pathological changes in neural tissues as in Alzheimer disease [9].

The major challenge of DKI in abdominal imaging relates to the difficulty in obtaining sufficient signal-to-noise ratio (SNR) at high b-values [10]. A parameter optimization is necessary to prevent low SNR, inherent in the diffusion technique, from impacting the result of key diffusion parameters. Strategies that may be used to increase SNR include imaging at a higher field strength (3.0 T); minimizing echo time (< 100 ms); increasing the number of signals acquired, which must be balanced against the resulting increase in imaging time.

Recently, there have been several articles on abdominal imaging demonstrating the feasibility of DKI and indicating that a DKI model may have added value in lesion or normal tissue characterization of liver and prostate [11, 12]. In particular, a recently published study by Ding et al. [13] has indicated that DKI is feasible in malignant and benign renal tumors. While promising results were obtained, i.e.,the MD values of CCRCCs were higher, while MK values were lower than those of benign renal tumors. However, their study did not include other DKI parametric results, i.e., FA,KA and RK. The influence of the SNR on the DKI results was not evaluated. Morover, small number of patients in benigne renal tumors (12 fat poor angiomyolipomas and two renal oncocytomas). In our study,we retrospectively investigated the imaging features of 86 cases of benign and malignant renal tumors with DKI, while the image SNRs were ensured.

## Methods

### Dataset

This retrospective study was approved by our institutional review board and written informed consent from all subjects was obtained before the study. Ninety-six renal tumors were confirmed by pathology and immunohistochemistry from January 2014 to January 2020. We searched the electronic medical record to identify 66 patients with clear cell renal cell carcinoma (CCRCC), 13 patients with renal angiomyolipoma with minimal fat (RAMF) and 7 patients with renal oncocytoma (RO) were examined who underwent 3.0-T kidney MRI. During this time, preoperative 3.0 T MRI of the renal tumor was routinely performed at our institution and included a DKI sequence.

### Magnetic resonance imaging (MRI)

MRI examinations were performed with a 3.0-T MR scanner (GE Signa EXCITE HD, Milwaukee, WI, USA) using a 6 channel array body coil and a 24 channel phased array spine coil integrated into the scanner table. For DKI, a single shot echo-planar imaging (EPI) sequence was applied in the axial plane using respiratory triggering via a respiratory belt with 3 b values (0, 500, 1000s/mm^2^), 30 diffusion directions and 8 signal averages. The other imaging parameters were as follows: 24 axial slices covering both kidneys; echo time (TE) = 59.2 ms, repetition time (TR) = 5000 ms, number of excitations (NEX = 2), matrix = 192 × 192, field of view (FOV) = 400 mm. ASSET as a parallel imaging method was applied with an acceleration factor of 2.

### Image analysis

**Fig. 1:**
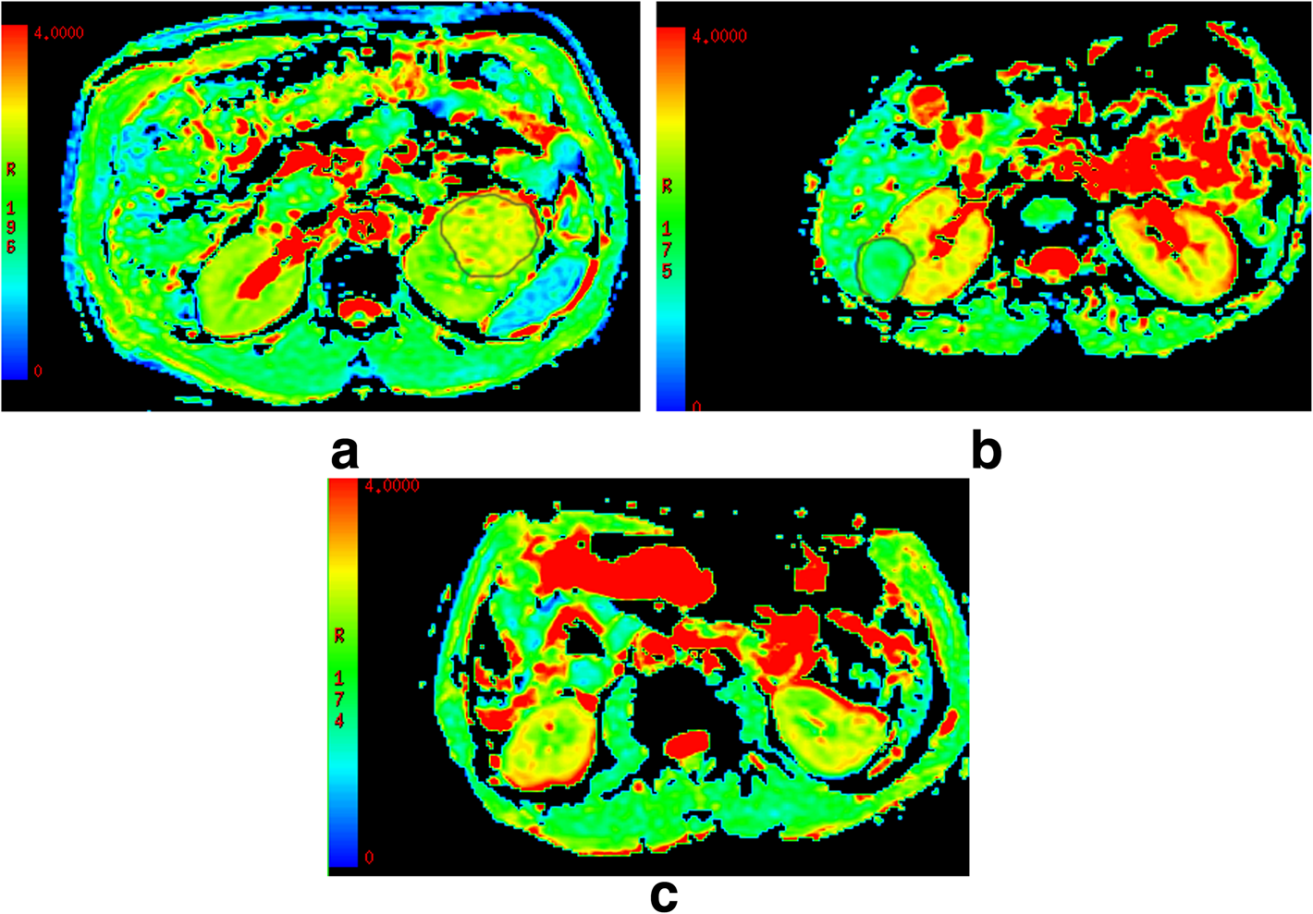
**a**-**c**: MD features of CCRCC **a**, AML **b** and RO **c**, respectively. MD regions of interest (outlined in black on MD images) for CCRCC and AML. A significant higher value was shown in CCRCC (3.08 ± 0.23) than the rest renal tumors (2.93 ± 0.30 for RO, 1.52 ± 0.24 for AML, *P* < 0.05)

Acquired images were transferred to an off-line workstation for processing. Before DKI quantification, image co-registration and smoothing were performed using automated image registration (AIR) software 4.6.4. All DWIs were first co-registered to the b0 image using the affine model. Then, registered DWIs with b values of 500 and 1000 s/mm^2^ were averaged over 30 diffusion-encoding directions. Afterwards, the two averaged DWIs were co-registered to the b0 image using the affine model, and the registered averaged DWIs were set as a reference volume for further registrations. Finally, the initial DWIs with b values of 500 and 1000 s/mm^2^ were co-registered to the corresponding reference volume using a non-rigid model. With our DKI protocol, we obtained parametric maps related to diffusional kurtosis: MD, FA, MK, KA and RK. The assessment of renal tumor and ROI positioning was conducted by a radiologist (with 5 years of clinical experience in interpreting MR images). ROIs of the mass was drawn around the most solid part of each tumor on T2 signal intensity maps using Image J (National Institutes of Health, Bethesda, MD, USA) in axial slices at five representative slices and were simultaneously copied to MD (Fig. 1a, b), FA, MK, KA and RK maps, respectively.The tumor area with the lower T2 signal intensity was selected as the most solid part for heterogeneous tumors. The areas with strong hyperintensity on T2WI were excluded as indicating necrotic tissues. These ROIs were selected on the basis of visual inspection of the parametric maps and the averaged values of the five slices were used for final analysis..

The influence of the SNR on the DKI results was evaluated on b = 0, 500 and 1000s/mm^2^ images. SNR was calculated by dividing the mean signal intensity within the ROI by the standard deviation (SD) of the background noise SNR = S/SD.

### Statistical analysis

Statistical analysis was undertaken using SPSS version 17.0 statistical software (SPSS, Chicago, IL, USA). Numeric data were expressed as means and standard deviations (±SD). Evaluated DKI parameters were compared with four tumor types by applying analysis of variance (ANOVA) and post-hoc test (Tukey). Values of *P* < 0.05 were considered statistically significant.

## Results

### Population demographics

Ninety-two renal tumors were enrolled in this study. Six cases were excluded from the study because of intense motion artifacts or incomplete acquisition of all sequences ascribed to a long acquisition time. Eighty-six renal tumors successfully completed the scans (53 men and 33 women; mean age ± standard deviation, 53.6 ± 8.6 years; age range, 39–69 years; tumor diameters, ranged from 4.8 to 12.6 cm, mean diameter, 6.2 ± 3.1 cm).

### Image quality assessment

SNRs were 31.9 for b = 0 s/mm^2^, 14.3 for b = 500 s/mm^2^ and 9.3 for b = 1000 s/mm^2^ images of the representative case.

### DKI parameters of the renal tumors

**Fig. 2:**
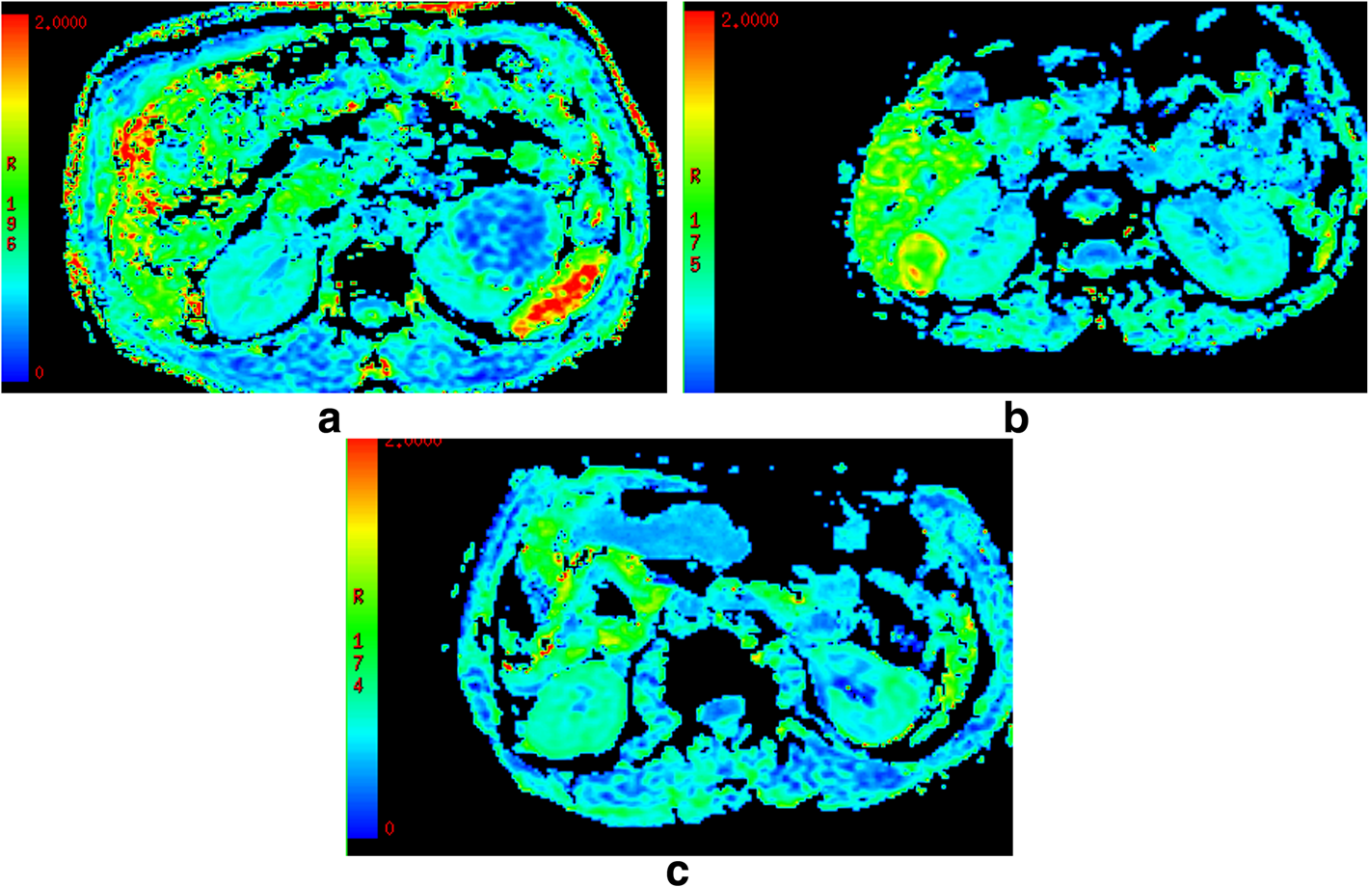
**a**-**c**: MK features of CCRCC **a**, AML **b** and RO **c**, respectively. A significant higher value was shown in AML (1.32 ± 0.16) than the rest renal tumors (0.81 ± 0.08 for RO, 0.43 ± 0.08 for CCRCC, *P* < 0.05)

**Fig. 3:**
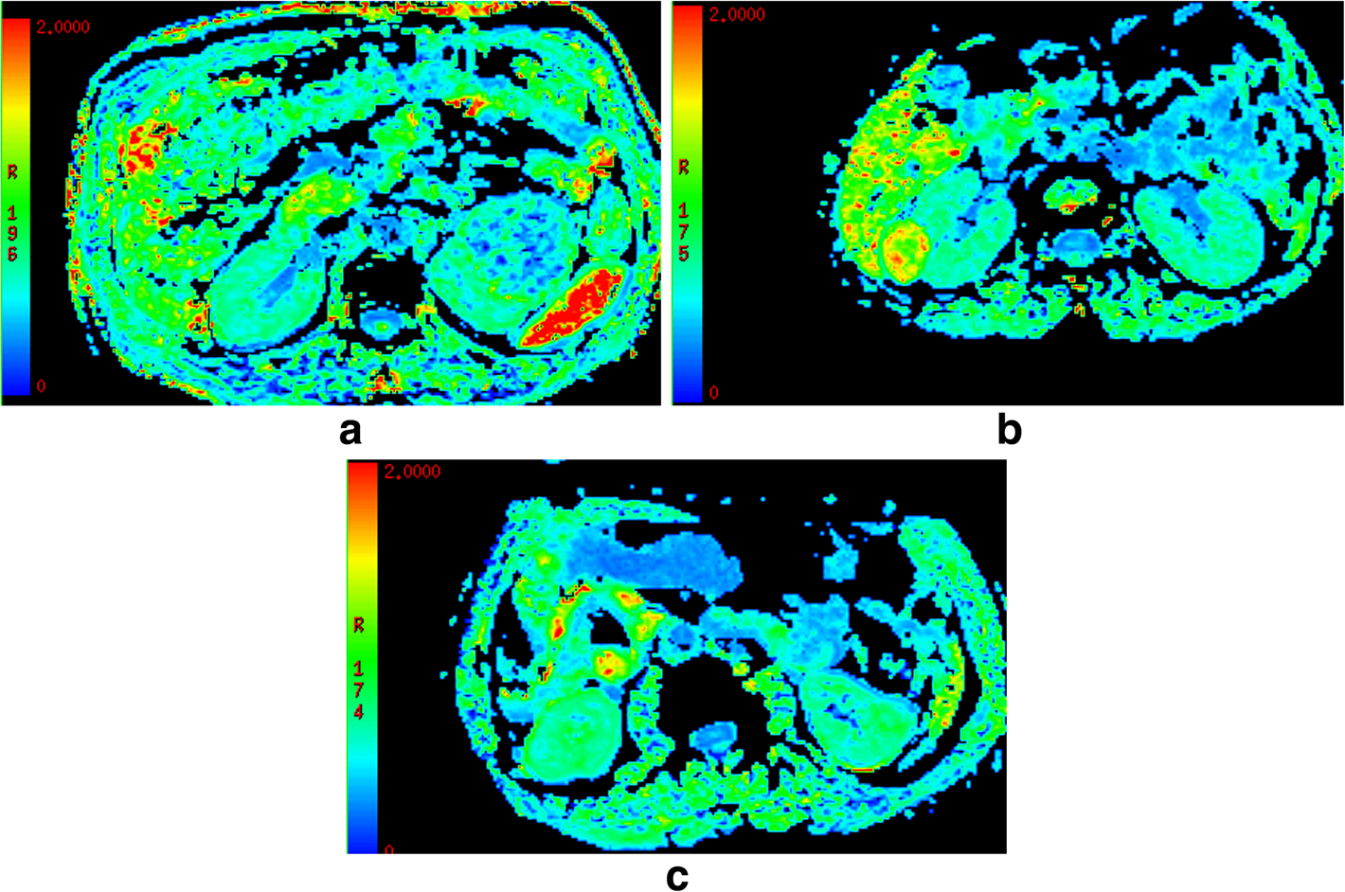
**a**-**c**: KA features of CCRCC **a**, AML **b** and RO **c**, respectively. A significant higher value was shown in AML (1.42 ± 0.23) than the rest renal tumors (0.86 ± 0.16 for RO, 0.57 ± 0.16 for CCRCC, *P* < 0.05)

**Fig. 4:**
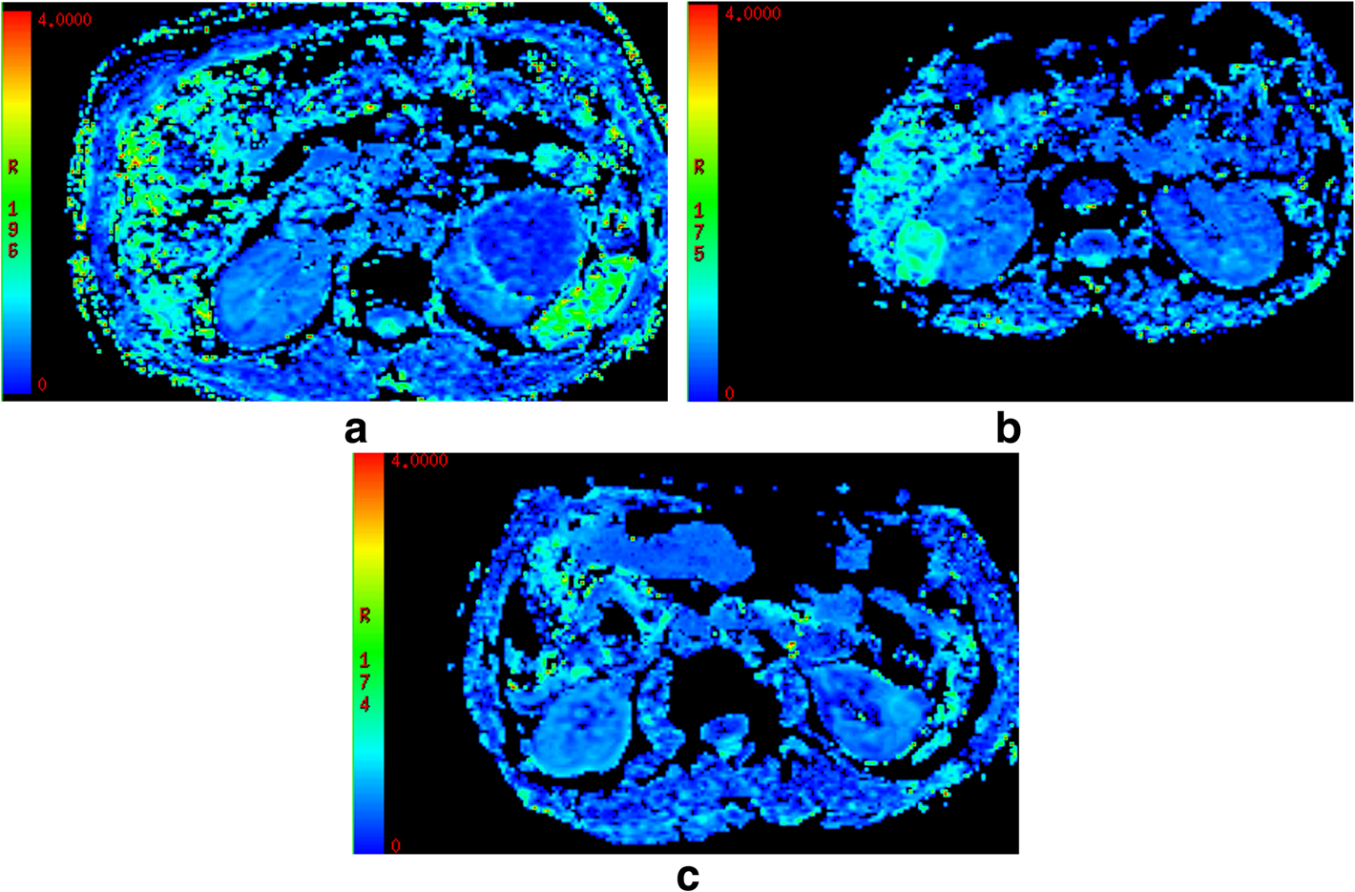
**a**-**c**: RK features of CCRCC **a**, AML **b** and RO **c**, respectively. A significant higher value was shown in AML (1.41 ± 0.29) than the rest renal tumors (0.69 ± 0.08 for RO, 0.37 ± 0.11 for CCRCC, *P* < 0.05)

DKI parameters of the four renal tumors are shown in Table 1. For MD (Fig. 1), a significant higher value was shown in CCRCC (3.08 ± 0.23) than the rest renal tumors (2.93 ± 0.30 for RO, 1.52 ± 0.24 for AML, *P* < 0.05). The MD values were higher for RO than for AML (2.93 ± 0.30 vs.1.52 ± 0.24, P < 0.05), while comparable MD values were found between CCRCC and RO (3.08 ± 0.23 vs. 2.93 ± 0.30, *P* > 0.05). For MK(Fig. 2), KA(Fig. 3) and RK(Fig. 4), a significant higher value was shown in AML (1.32 ± 0.16, 1.42 ± 0.23, 1.41 ± 0.29) than CCRCC (0.43 ± 0.08, 0.57 ± 0.16, 0.37 ± 0.11) and RO (0.81 ± 0.08, 0.86 ± 0.16, 0.69 ± 0.08) (*p* < 0.05). The MK, KA and RK values were higher for RO than for CCRCC (0.81 ± 0.08 vs. 0.43 ± 0.08, 0.86 ± 0.16 vs. 0.57 ± 0.16, 0.69 ± 0.08 vs. 0.37 ± 0.11, *P* < 0.05).

**Table 1:** The DKI parameters in CCRCC, AML and RO

Type	MD	FA	MK	KA	RK
CCRCC	3.08 ± 0.23	0.26 ± 0.07	0.43 ± 0.08	0.57 ± 0.16	0.37 ± 0.11
AML	1.52 ± 0.24	0.23 ± 0.05	1.32 ± 0.16	1.42 ± 0.23	1.41 ± 0.29
RO	2.93 ± 0.30	0.16 ± 0.04	0.81 ± 0.08	0.86 ± 0.16	0.69 ± 0.08
P	< 0.05	> 0.05	< 0.05	< 0.05	< 0.05

Note: *CCRCC* clear cell renal cell carcinoma, *RAMF* renal angiomyolipoma with minimal fat, *RO* renal oncocytoma, *MD* Mean Diffusivity, *FA* fractional Anisotropy, *MK* mean kurtosis (MK), *KA* kurtosis anisotropy, *RK* radial kurtosis

The MD values were higher for RO than for AML (2.93 ± 0.30 vs.1.52 ± 0.24, *P* < 0.05), while comparable MD values were found between CCRCC and RO (3.08 ± 0.23 vs. 2.93 ± 0.30, *P* > 0.05)

The MK, KA and RK values were higher for RO than for CCRCC (0.81 ± 0.08 vs. 0.43 ± 0.08, 0.86 ± 0.16 vs. 0.57 ± 0.16, 0.69 ± 0.08 vs. 0.37 ± 0.11, *P* < 0.05)

Using MD values of 2.86 as the threshold value for differentiating CCRCC from RO and AML, the best result obtained had a sensitivity of 76.1%, specificity of 72.6%. Using MK, KA and RK values of 1.19,1.13 and 1.11 as the threshold value for differentiating AML from CCRCC and RO, the best result obtained had a sensitivity of 91.2, 86.7, 82.1%, and specificity of 86.7, 83.2, 72.8%.

### T2-weighted imaging signal intensity

**Fig. 5:**
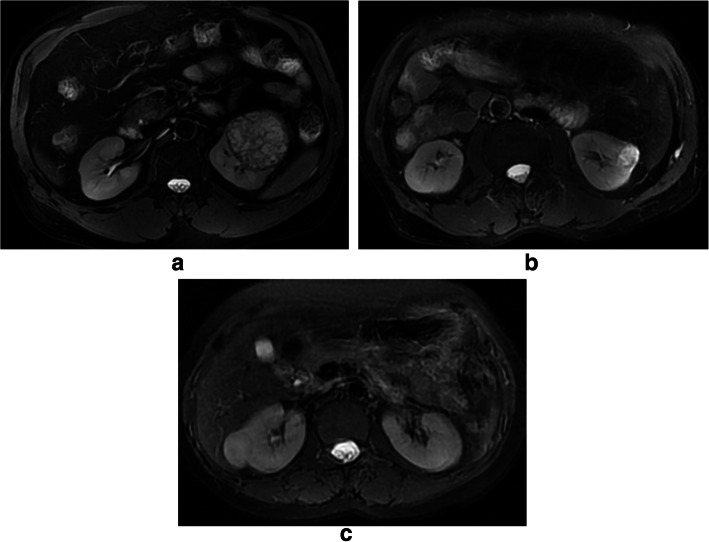
**a**-**c:** T2-weighted imaging signal intensity. CCRCC **a** and RO **b** showed hyperintense whereas AML **c** showed hypointense on T2-weighted imaging

CCRCC (Fig. 5a) and RO (Fig. 5b) showed hyperintense whereas AML (Fig. 5c) showed slightly hypointense on T2-weighted imaging.

## Discussion

Recently, there have been several articles on abdominal imaging demonstrating the feasibility of DKI and indicating that a DKI model may have added value in lesion or normal tissue characterization of liver and prostate [14, 15]. Rosenkrantz [11] applied non-Gaussian DWI for a better characterization of diffusion processes in the prostate. However, considering the decreased accuracy of the fitting equation and the relatively low SNR resulting from the use of higher b values, the maximum b value must be cautiously chosen. Steven et al. [16] have reported that the choice of the maximum b value should be based on the diffusivity and kurtosis value of the studied tissues. In normal human brain, it is assumed that D ≈ 1 × 10^− 3^ mm^2^/s and K ≈ 1, and the recommended DKI protocol for brain uses 2000 s/mm^2^ as the maximum b value [17]. Again, Rosenkrantz [15] applied the kurtosis model in the prostate at maximal diffusion strength of 800 s/mm^2^. The apparent diffusion coefficients in normal human kidney (both cortex and medulla) are generally around 2 × 10^− 3^ mm^2^/s or higher, which is about twice the value in the brain. Because of the low SNR at high b-values in abdominal DKI and the above mentioned reasons [18, 19], the choice of 30 diffusion directions and b-values up to 1000 s/mm^2^ seems appropriate for renal DKI.

Previous studies reported a better characterization of tissue microstructure with kurtosis measurements in the brain [20]. Therefore one can expect that DKI parameters might differ in renal tumors. Consistently MK of the cortex was constantly higher than that of the medulla in all four sequences [21]. While the present study concentrates on the non-Gaussian analysis of the biological tissue microstructure using the kurtosis method, various groups did report results based on other higher diffusion models [22].

Pentang et al. [13] has applied DKI in human kidney, and the maximum b value adopted was 600 s/mm^2^, with the fitted data showing that the departure of the diffusion signal from mono exponential behavior could be observed when b values reached approximately 600–800 s/mm^2^. They could show significant differences in FA and MK values of the cortex and medulla healthy volunteers. Comparing MKvalues with various renal diseases may help to evaluate the clinical significance of renal kurtosis values and the role of the renal DKI. For instance in renal cancer, DKI may provide additional diagnostic information.

In our study, we could show that b-values in the range of about 0 to 1000 s/mm^2^, with 30 diffusion encoding directions are sufficient in abdominal DKI to observe the departure of the diffusion signal from mono-exponential behavior. Strictly limited amount of topics involving DKI method in the study of renal tumors. In our study, the MD values were higher for CCRCC and RO, lower for AML. Tissue free water contents and structures can influence MD [23]. Increase in MD due to micronecrosis or altered viscosity of the medium possibly counterbalances decreased MD values in CCRCC and RO. CCRCC and RO is rich in lipid content of its cells; cholesterol, neutral lipids, and phospholipids are abundant on pathology. In our MR cases, we also noted hyperintense signal on T2 weighted imaging for CCRCC and RO. There was a good consistency between microscopic appearances of CCRCC, RO and T2WI characteristics. In the present study, using MD values of 2.86 as the threshold value for differentiating CCRCC from RO and AML, the best result obtained had a sensitivity of 76.1%, specificity of 72.6%.

Our findings of FA data showed no significant difference in different four renal tumor types, although there was a tendency in our study toward higher FA values for CCRCC that was not significant. Huang [23] reported higher FA for medulla than for cortex that might be caused by the radially oriented vessels, tubules and collecting ducts in the medulla. Inoue et al. [24] reported higher FA values for high-grade gliomas than for low-grade gliomas. Altogether, the value of FA measurements remains controversial.

Tissue structure can influence MK and KA [25]; therefore, these two metrics are not completely independent from each other, although MK and KA can be used to test for different aspects of diffusion. The increasing MK and KA values likely are caused by increasing viscosity in the tissue. In our cases, AML showed greater MK and KA values, CCRCC showed lowest values, and RO showed intermediate values. In our MR cases, we noted slight hypointense signal on T2 weighted imaging of AML. There was a good consistency between microscopic appearances of AML and T2WI characteristics. These reasons caused restriction of water diffusion deviates the diffusion distribution from the Gaussian form. This might favor the interpretation of increased MK and KA values being caused by increased cellular density [26]. Furthermore, it should be noted that the presence of the rich vasculature will complicate the interpretation of the diffusion pattern in the kidney [27]. Although the impact of renal blood flow or vasculature on kurtosis values of the kidney has not been determined.In the present study, using MK, KA values of 1.19,1.13 as the threshold value for differentiating AML from CCRCC and RO, the best result obtained had a sensitivity of 91.2, 86.7%, and specificity of 86.7, 83.2%.

Based on the viscosity that are greater in microstructure generally have a larger number of diffusion barriers, causing water diffusion to deviate more from a Gaussian distribution, a higher RK value typically implies a greater viscosity [28]. As illustrated in our study, RK of AML is higher than those of the CCRCC and RO, compatible with the knowledge that the AML have a greater viscosity and the restriction of water diffusion by the walls of hemorrhage or haemosiderin deposition.

The main limitation to our study is that the small number of patients in each type of renal tumors. Recommend further studies with larger population to validate the results of our study. Furthermore, respiratory movements of the kidney are mainly in a cranio-caudal direction, and do not always coincide with the abdominal wall movements. Finally, we used 1000s/mm^2^ as the maximum b value in this study, which is much smaller than the recommended 2000 s/mm^2^ for brain. However, considering the decreased accuracy of the fitting equation and the relatively low SNR resulting from the use of higher b values, we hope that the adopted b values, as an initial attempt, could be of reference value to the protocol settings of further DKI studies in the kidney. In the present study, using RK values of 1.11 as the threshold value for differentiating AML from CCRCC and RO, the best result obtained had a sensitivity of 82.1%, and specificity of 72.8%.

## Conclusions

In conclusion, this study’s results demonstrate significant differences in DKI between benign and malignant renal tumors. This new technique potentially can be used as another noninvasive biomarker for renal tumor type’s differential diagnosis.

## Data Availability

The image dataset is available at the Medical Imaging, Clinical Medical College, Yangzhou University, Yangzhou, China.

## Abbreviations

CCRCC: Clear cell renal cell carcinoma
RAM: Renal angiomyolipoma with minimal fat
RO: Renal oncocytoma
DKI: Diffusion kurtosis imaging
MD: Mean Diffusivity
FA: Fractional Anisotropy
MK: Mean kurtosis
KA: Kurtosis anisotropy
RK: Radial kurtosis
